# Risk factors and injury patterns of e-scooter associated injuries in Germany

**DOI:** 10.1038/s41598-022-25448-z

**Published:** 2023-01-13

**Authors:** Holger Kleinertz, Annabelle Volk, Dimitris Dalos, Rico Rutkowski, Karl-Heinz Frosch, Darius M. Thiesen

**Affiliations:** 1 Department of Trauma and Orthopaedic Surgery, University Medical Center Hamburg-Eppendorf, Martinistr. 52, 20246 Hamburg, Germany; 2 UKE Athleticum-Center for Athletic Medicine, University Medical Center Hamburg-Eppendorf, Hamburg, Germany; 3 Institute of Interdisciplinary Exercise Science and Sports Medicine, MSH Medical School Hamburg, Hamburg, Germany; 4 Department of Oral and Maxillofacial Surgery, University Medical Center Hamburg-Eppendorf, Hamburg, Germany; 5 Department of Trauma Surgery, Orthopaedics, and Sports Traumatology, BG Hospital Hamburg, Hamburg, Germany

**Keywords:** Disease prevention, Health care economics, Patient education

## Abstract

Since the introduction of widely available e-scooter rentals in Hamburg, Germany in June of 2019, our emergency department has seen a sharp increase in the amount of e-scooter related injuries. Despite a rising number of studies certain aspects of e-scooter mobility remain unclear. This study examines the various aspects of e-scooter associated injuries with one of the largest cohorts to date. Electronic patient records of emergency department admissions were screened for e-scooter associated injuries between June 2019 and December 2021. Patient demographic data, mechanism of injury, alcohol consumption, helmet usage, sustained injuries and utilized medical resources were recorded. Overall, 268 patients (57% male) with a median age of 30.3 years (IQR 23.3; 40.0) were included. 252 (94%) were e-scooter riders themselves, while 16 (6%) were involved in crashes associated with an e-scooter. Patients in non-rider e-scooter crashes were either cyclists who collided with e-scooter riders or older pedestrians (median age 61.2 years) who tripped over parked e-scooters. While e-scooter riders involved in a crash sustained an impact to the head or face in 58% of cases, those under the influence of alcohol fell on their head or face in 84% of cases. This resulted in a large amount of maxillofacial soft tissue lacerations and fractures. Extremity fractures and dislocations were more often recorded for the upper extremities. This study comprises one of the largest cohorts of e-scooter associated injuries to date. Older pedestrians are at risk to stumble over parked e-scooters. E-scooter crashes with riders who consumed alcohol were associated with more severe injuries, especially to the head and face. Restricted e-scooter parking, enforcement of drunk driving laws for e-scooters, and helmet usage should be recommended.

## Introduction

Since beginning in California, USA in 2017, ride-sharing systems for electrically powered scooters (e-scooters) have been introduced in several countries^1,2^. Consequently, emergency departments (ED) around the globe have registered a rising number of e-scooter associated injuries^1,3–6^. The e-scooter sharing system was introduced in Germany in June of 2019, with several providers making this mode of transportation easily available for a broad range of customers. Riders must be a minimum of 14 years old, no license is required, the maximum speed is set to 20 km/h, and the legal alcohol limits are equivalent to those for driving a car^7^. With the increasing popularity and number of e-scooters available, discussions about safety, responsible use, and considerate driving and parking have attracted media attention. Cities such as Hamburg, Germany have set up voluntary agreements with e-scooter providers to successfully integrate e-scooters into urban mobility concepts^8^.

One of the first and currently the largest studies focusing on e-scooter associated injury patterns pointed out a high percentage (40%) of head injuries after e-scooter related crashes, with less than 5% of riders wearing a helmet. Furthermore, the authors describe a high fracture incidence, with 31% of e-scooter riders sustaining a fracture of any kind^1,9^. While e-scooter crashes under the influence of alcohol were rather rare in the United States, studies from Europe and New Zealand showed alcohol intoxication in around one third of e-scooter riders presenting to the ED^3,4,10,11^. The high percentage of maxillofacial injuries recorded while under the influence of alcohol led Blomberg et al. to conclude that “e-scooter riders are young adults who fall on their faces, often under the influence of alcohol or drugs”. This group also was the first from Europe to raise concerns about non-riders being injured by e-scooter riders or parked e-scooters^3^.

While some aspects of e-scooter related trauma have been well documented, others remain unclear. The influence of alcohol on e-scooter associated injuries has been described, but it has not been established which injuries are associated with drunk riding. Further, the broad range of extremity injuries has not yet been described in detail. Lastly, the impact of e-scooter riders and e-scooters parked on sidewalks on pedestrians and cyclists, plus the associated injuries, need to be further investigated.

This study presents one of the largest cohorts of e-scooter-related injuries to date. Our aim is to deliver focused evidence to provide recommendations for the safe operation of e-scooters and injury prevention programs.

## Materials and methods

A retrospective analysis was performed by searching the electronic medical records of all patients presenting to the ED or outpatient orthopaedic clinic of an urban German level 1 trauma center between June 2019 and December 2021. The medical records were screened for the keywords “scooter” and the corresponding German term “Roller”. The medical documentation was analyzed and all patients who sustained an injury due to the usage of an e-scooter or those associated with an e-scooter were included.

Patient age and sex were recorded. Details about the mode of arrival, time and date of presentation, as well as triage code according to the Manchester Triage System were assessed^12^. Moreover, the mechanism of injury was recorded and categorized, as well as alcohol consumption and helmet usage. Alcohol consumption was based on breath-testing, blood alcohol, patient reported relevant alcohol consumption, or physician perception. Injuries obtained, medical disciplines involved, and utilized diagnostic resources were recorded. Additionally, treatment in the ED, surveillance and vital sign monitoring requirement, length of inpatient stay, as well as necessary surgeries were documented.

### Statistical analysis

This is a descriptive study without alpha error adjustment that reports a patient cohort from a sample population in Hamburg, Germany. ﻿Preparation of graphs and statistical analyses were performed using GraphPad PrismVersion 9 (GraphPad Software, La Jolla, USA). Continuous variables were summarized by the median and interquartile range. The Shapiro–Wilk test was used for normality testing. Depending on whether a normal distribution was present, the t-test or Mann–Whitney U test was applied.

### Ethics approval and consent to participate

The study was approved by the local ethics committee of the medical board in Hamburg, Germany (Ethik-Kommission der Ärztekammer Hamburg, PV7262) and conducted in accordance with the ethical standards laid out by the 1964 Declaration of Helsinki. Patient consent was waived by the local ethics committee of the medical board in Hamburg, Germany (Ethik-Kommission der Ärztekammer Hamburg, PV7262).

## Results

### Epidemiology and injury characteristics

A total of 268 patients with e-scooter associated injuries were included in the present study. The median age was 30.3 (IQR 23.3; 40.0) years, of which 153 (57%) were male. 252 (94%) cases were e-scooter riders, with a mean age of 29.9 years (IQR 23.1; 39.6) and 72% of patients between 18 and 40 years of age. Non-rider e-scooter associated injuries accounted for 16 (6%) cases (Table 1).

**Table 1:** Epidemiology, mechanism and cause of injury and mode of presentation of e-scooter riders and non-e-scooter riders.

	Riders [%]	Non-riders [%]	Total [%]
**Total n**	252	16	268
**Median Age (IQR)**	29.9 (23.1;39.6)	37.2 (29.1;70.0)	30.3 (23.3;40.0)
Age< 18	16 [6]	0 [0]	16 [6]
18–25	71 [28]	3 [19]	74 [28]
26–40	112 [44]	7 [44]	119 [44]
41–64	50 [20]	2 [13]	52 [19]
≥ 65	3 [1]	4 [25]	7 [3]
male [%]	145 [58]	8 [50]	153 [57]
**Mechanism of injury**			
Fall, not otherwise specified	206 [82]	–	189 [71]
Collision with stationary object	13 [5]	7 [44]	24 [9]
Collision with moving object	19 [8]	8 [50]	27 [10]
Other	14 [6]	1 [6]	28 [10]
**Time of injury**			
7 AM to 3 PM	46 [18]	7 [44]	53 [20]
3 PM to 11 PM	80 [32]	4 [25]	84 [31]
11 PM to 7 AM	98 [39]	3 [19]	101 [38]
Not available	28 [11]	2 [13]	30 [11]
**Mode of presentation**			
Walk in	141 [56]	5 [31]	146 [54]
Ambulance/Paramedics	93 [37]	11 [69]	104 [39]
Emergency physician	9 [4]	–	9 [3]
Not available	9 [4]	–	9 [3]
**Triage according to Manchester-System [12]**			
1	12 [5]	0	12 [4]
2	34 [13]	3 [19]	37 [14]
3	63 [25]	3 [19]	66 [25]
4	71 [28]	7 [44]	78 [29]
5	50 [20]	2 [13]	52 [19]
Not available	22 [9]	1 [6]	23 [9]
**Under the influence of alcohol**	83 [33]	2 [13]	85 [32]
**Helmet use**	2 [1]	Not applicable	5 [2]
**Impact to head/face**			
Yes	147 [58]	11 [69]	158 [59]
No	87 [35]	3 [19]	90 [34]
Not available	18 [7]	2 [13]	20 [7]

For e-scooter riders, falls were the main mechanism of injury in 206 (82%) cases. Patients regularly reported uneven pavement (e.g., sidewalk, cobblestone) or a slippery road (e.g., rain, foliage) to have caused the fall. Collisions with stationary or moving objects were less frequent (Table 1). The 13 (5%) patients who collided with a stationary object reported garbage cans, streetlamps, and poles on the walkway. Of the 19 (8%) patients who had a collision with a moving object, 14 reported a car, two a truck, two another e-scooter and one a bicycle rider. The 14 (6%) e-scooter riders who reported other mechanisms of injury sustained a cut at the ankle when pushing of the ground to accelerate, bumped against parts of the scooter when braking or hurt themselves jumping of a riding e-scooter.

**Figure 1:**
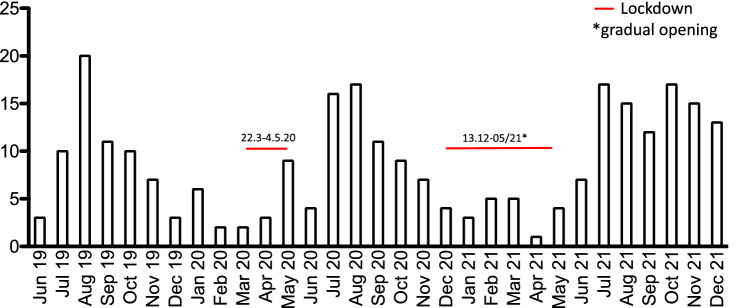
Graph depicting e-scooter associated ED presentations and effect of Covid-19 lockdown on number of presentations.

139 (55%) e-scooter crashes happened between Friday and Sunday, 98 (39%) presented between 11 PM and 7 AM (Table 1). The number of e-scooter crashes peaked during the summer months, with spring 2020 and 2021 showing a low incidence, possibly due to COVID-19 pandemic lockdown measures in Germany (Fig. 1).

141 (56%) of e-scooter riders presented as “walk-ins” to the ED, 93 (37%) were brought in by ambulance/paramedics, and nine (4%) by an emergency physician. According to the Manchester triage System^12^, most of the e-scooter riders were triaged as urgent, standard, or non-urgent, while 18% were triaged as needing immediate or very urgent care.

83 (33%) e-scooter injuries were sustained under the influence of alcohol. An impact to the head or face was recorded for 147 (58%) of all e-scooter riders. Two (1%) e-scooter riders wore a helmet (Table 1).

**Figure 2:**
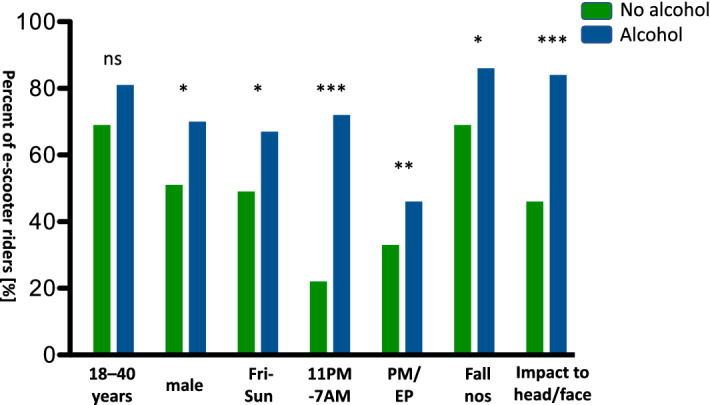
Bar graph depicting sober e-scooter riders vs. those under the influence of alcohol. Depicted as bars in percent of total of those aged between 18 and 40 years, male sex, crashes between Friday and Sunday, crash time between 11 PM and 7 AM, mode of presentation by ambulance/paramedics (PM) or emergency physician (EP), crash mechanism "fall, not otherwise specified (nos)” and impact to head and face. Statistical analysis with Mann–Whitney test (*p < 0.05, **p < 0.01, ***p < 0.001).

The 83 e-scooter riders who were under the influence of alcohol were more often male (*p* = 0.0136) and the crashes occurred more often on weekends (*p* = 0.0148) between 11 pm and 7 am (*p* < 0.0001). E-scooter riders under the influence of alcohol were more often brought in by ambulance/ paramedics or an emergency physician (*p* = 0.0007). While 84% of e-scooter riders under the influence of alcohol suffered an impact to their head or face, those who were not under the influence of alcohol suffered an impact to the face or head in 46% of cases (*p* < 0.0001) (Fig. 2). Alcohol breath testing or blood alcohol levels were acquired in 25 cases with the median alcohol level being 1.5‰ (Range 0.7‰ to 2.9‰).

The 16 (6%) non e-scooter riders were significantly (*p* < 0.0186) older than the e-scooter riders, with a median age of 37.2 (IQR 29.1; 70.0). Among the non-riders, eight cyclists (50%) collided with an e-scooter rider. Three out of eight (38%) cyclists who collided with an e-scooter wore a helmet. Falls over e-scooters accounted for seven (44%) cases and one (6%) case was an e-scooter passenger. The median age of those seven patients who tripped over an e-scooter was 61.2 (IQR 33.9; 83.3) years and three were 80 years and above. Non-riders presented as “walk-ins” (31%) or were brought in by ambulance/paramedics (69%). Among the non-riders, no patient was triaged as needing immediate and 3 (19%) as needing very urgent care (Table 1).

### Injury patterns

**Figure 3:**
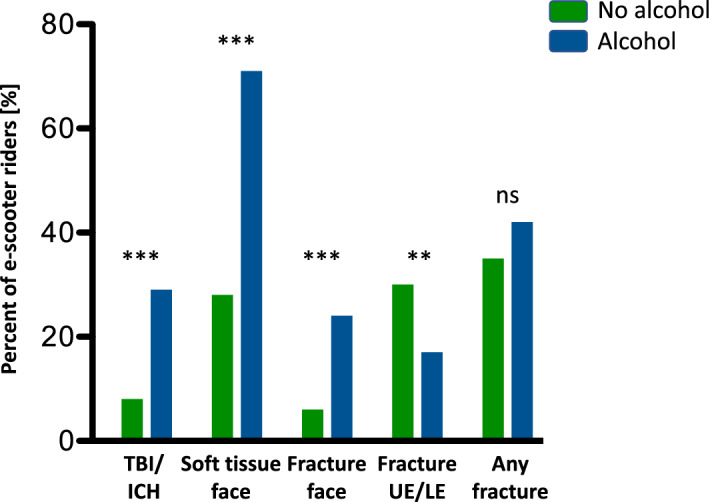
Bar graph depicting sober e-scooter riders vs. those under the influence of alcohol. Depicted as bars in percent of total of those who sustained a traumatic brain injury (TBI) or intracranial hemorrhage (ICH), soft tissue injury to the face, maxillofacial fracture, upper or lower extremity fracture (UE/LE), and any fracture. Statistical analysis with Mann–Whitney test (*p < 0.05, **p < 0.01, ***p < 0.001).

E-scooter riders sustained traumatic brain injuries (TBI) in 31 (12%) cases and had an intracranial hemorrhage (ICH) in six (2%) cases. Soft tissue injuries to the face were found in 107 (42%) and maxillofacial fractures in 30 (12%) cases. Dental injuries were recorded for 45 (18%) e-scooter riders (Table 2). E-scooter riders under the influence of alcohol were found to have a significantly higher risk of TBI/ICH (29% vs. 8%, *p* < 0.0001), as well as soft tissue injuries to the face (71% vs. 28% *p* < 0.0001) and maxillofacial fractures (24% vs. 6% *p* < 0.0001) when compared to sober riders (Fig. 3).

**Table 2:** Injury patterns of e-scooter riders and non-e-scooter riders according to body regions.

	Riders no [%]	Non-riders no [%]	Total no [%]
**Total n**	252	16	268
**Head**			
Contusion	34 [13]	4 [25]	38 [14]
Traumatic brain injury (TBI)	31 [12]	1 [6]	32 [12]
Intracranial hemorrhage (ICH)	6 [2]	–	6 [2]
Soft tissue	7 [3]	2 [13]	9 [3]
Fracture	4 [2]	–	4 [1]
**Face**			
Contusion	38 [15]	1 [6]	39 [15]
Soft tissue	107 [42]	5 [31]	112 [42]
Fracture	30 [12]	1 [6]	31 [12]
Teeth	45 [18]	2 [13]	47 [18]
**Thoracic**			
Contusion	12 [5]	1 [6]	13 [5]
Fracture	1 [0]	1 [6]	2 [1]
**Abdominal**			
Liver laceration	1 (0)	**–**	
**Spine**			
Contusion/Strain/Sprain	13 [5]	–	13 [5]
Fracture	1 [0]	–	1 [0]
**Pelvic contusion**	3 [1]	–	3 [1]
**Fracture upper extremity**	42 [17]	2 [13]	44 [16]
**Fracture lower extremity**	18 [7]	–	18 [7]
**Cases with a fracture**	94 [37]	4 [25]	98 [37]
**Cases with a dislocation**	14 [6]	2 [13]	16 [6]

Injuries to the thorax, abdomen, spine, and pelvis were rare. One fractured rib, one fracture of the third thoracic vertebrae, and one liver laceration were seen in e-scooter riders. Overall, 94 (37%) fractures and 14 (6%) joint dislocations were recorded for e-scooter riders (Table 2). A total of 67 (27%) e-scooter riders sustained a fracture or joint dislocation of the upper or lower extremities (Table 3). While the overall number of fractures did not differ among riders with and without alcohol consumption, those riders who consumed alcohol were found to have significantly more maxillofacial (*p* < 0.0001) but less extremity fractures (*p* = 0.0015) (Fig. 3).

**Table 3:** Serious Injuries of the extremities sustained by e-scooter riders.

Extremity injuries	
**Fracture/dislocation**	67 [27% of total]
- indication for surgery	38 [57%]*
	**n [surgery required]**
**Upper extremity**	49
Fracture	37
Dislocation	7
Dislocation and fracture	5
Clavicle	5 [4]
AC joint injury	4 [2]
Shoulder dislocation - 1 with fracture of greater tubercule, 1 with large Hill-sachs lesion - 2 with glenoid fracture	6 [3]
Proximal humerus	3 [0]
Radial head/neck	14 [2]
Olecranon	2 [2]
Wrist	3 [1]
Carpus	6 [2] (incl. 3 Scaphoid fractures)*
Hand and fingers	6 [4]
	1 indication due to soft tissue injury of the elbow
**Lower extremity**	18
Fracture	16
Dislocation	0
Dislocation and fracture	2
Patella	4 [3]
Patellar tendon rupture	1 [1]*
ACL	2 [1]*
Meniscus	2 [1]*
MCL	1 [1]*
Tibial plateau	5 [5]*
Lower leg	1 [1] open fracture
Ankle	7 [6]*
Toe	1 [1]
	3 indications due to soft tissue injuries of the knee and ankle

*total number of cases that required surgery is lower than overall indications due to two patients who had surgically addressed injuries of the upper and lower extremity and two patients with complex knee injuries with bone and soft tissue injury.

Severe upper extremity injuries, such as fractures or joint dislocations, were more common (*n* = 49; 73%) than lower extremity injuries (*n* = 18; 27%). The most common fracture of the upper extremity, with 14 cases, was a radial head or neck fracture, followed by fractures of the carpal and metacarpal bones/fingers, with six cases each. We found six shoulder dislocations, four accompanied by a fracture of the glenoid, greater tubercle, or a large Hill-Sachs lesion. Acromioclavicular joint dislocations were seen in four cases (Table 3).

The most common fractures of the lower extremity were ankle fractures with seven cases, of which six required surgery. Six fractures of the tibial plateau occurred, five of which required surgery. Of those six tibial plateau fractures, two were accompanied by a rupture or bony avulsion of the anterior cruciate ligament (ACL) and either a rupture of the medial collateral ligament (MCL) or the patellar tendon. One isolated ACL rupture and one bucket-handle meniscus tear were found. There were four patellar fractures, three of which required surgery, and one open tibial fracture also requiring surgery (Table 3).

Non-riders sustained a concussion, a maxillofacial fracture, and a serial rib fracture in one case (6%) each. Dental injuries and fracture dislocations could be found in two cases (13%). Further, four fractures (25%) and five soft tissue injuries to the face (31%) were diagnosed in non-riders (Table 2).

### Diagnostics, therapy and emergency department disposition

Most e-scooter riders (92%) were seen by an orthopaedic trauma surgeon in the ED. Patients not seen by an orthopaedic trauma surgeon had isolated injuries to the face that were primarily treated by a maxillofacial surgeon, the second most involved discipline. Maxillofacial surgeons were involved in the treatment of 125 (50%) e-scooter riders. Further, neurologic or neurosurgical expertise was required for 52 (21%) riders. Other disciplines were less frequently involved (Table 4).

**Table 4:** Involved disciplines, means of diagnostics and treatment received by e-scooter riders and non-e-scooter riders.

	Riders no [%]	Non-riders no [%]	Total no [%]
**Total n**	252	16	268
**Involved disciplines**			
Orthopaedic surgery	231 [92]	15 [94]	246 [92]
Maxillofacial surgery	125 [50]	7 [44]	132 [49]
Neurology/Neurosurgery	52 [21]	3 [19]	55 [21]
Others (General surgery, Ophthalmology, Otolaryngology, Internal Medicine)	34 [13]	2 [13]	36 [13]
**Standard radiograph**	154 [61]	9 [56]	163 [61]
**CT**	79 [31]	3 [19]	82 [31]
**CCT**	32 [13]	2 [13]	34 [13]
**MRI**	18 [7]	–	18 [7]
**Wound treatment**	105 [42]	6 [38]	111 [41]
Face	85 [34]	4 [25]	89 [33]
Other	20 [8]	2 [13]	22 [8]
**Cast/Brace**	73 [29]	5 [31]	78 [29]
**Disposition from ED**			
Outpatient	172 [68]	11 [69]	183 [68]
Direct admission	58 [23]	4 [25]	62 [23]
Secondary admission	22 [9]	1 [6]	23 [9]
Monitoring	42 [17]	2 [13]	44 [16]
ICU	–	–	–
**Days of median inpatient length of stay (IQR)**	2.0 (1.0;5.3)	1.5 (1.0;9.5)	2.0 (1.0–5.5)
**Indication for operative treatment**	62 [25]	3 [19]	65 [24]
Orthopaedic surgery	38 [15]	2 [13]	40 [15]
Maxillofacial surgery	24 [10]	1 [6]	25 [9]

**Figure 4:**
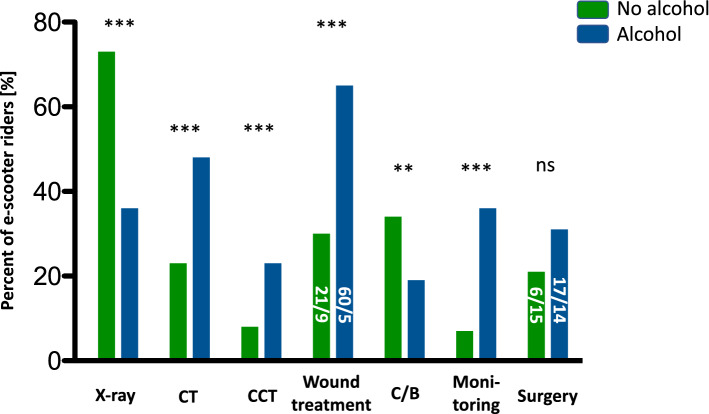
Bar graph depicting sober e-scooter riders vs. those under the influence of alcohol. Depicted as bars in percent of total of those who received a standard radiograph (X-ray), computed tomography (CT), cranial computed tomography (CCT), wound treatment, cast or brace (C/B), 24-h continuous vital sign monitoring, and surgery. Wound treatment and operations divided for maxillofacial surgeon (top) / orthopaedic surgeon (bottom) in percent. Statistical analysis with Mann–Whitney test (*p < 0.05, **p < 0.01, ***p < 0.001).

Regarding imaging, 154 (61%) e-scooter riders received standard radiographs (X-ray). Computed tomography (CT) scans were obtained from 79 (31%) riders. 32 (13%) e-scooter riders had a cranial CT (CCT) scan taken. 18 (7%) e-scooter riders received magnetic resonance imaging (MRI) (Table 4). While e-scooter riders under the influence of alcohol received significantly less X-ray’s (*p* < 0.001), CT or CCT scans were taken significantly more often (*p* < 0.0001 / *p* = 0.0007) (Fig. 4).

Surgical wound treatment was needed by 105 (42%) riders. The face was by far the most frequently injured region requiring surgical wound treatment. 73 (29%) e-scooter riders received a cast or brace (Table 4). E-scooter riders under the influence of alcohol received surgical wound treatment significantly more often (*p* < 0.0001) but required a cast or brace less often when compared to sober riders (*p* = 0.02). The increased requirement for wound treatment of those under the influence of alcohol was primarily due to lacerations of the face (Fig. 4).

Most e-scooter riders (68%) could be discharged from the ED with a referral for further outpatient treatment. Direct admission was indicated for 58 (23%) riders. Secondary admission for elective operative treatment was necessary for 22 (9%) e-scooter riders. No e-scooter rider was admitted to the intensive care unit (ICU) while 42 (17%) riders underwent continuous surveillance and vital sign monitoring for 24 hours (Table 4). Riders under the influence of alcohol required 24-h continuous vital sign monitoring significantly more often than those who were sober (*p* < 0.0001) (Fig. 4). Inpatient length of stay was 2.0 (IQR 1.0; 5.3) days for e-scooter riders (Table 4).

For all disciplines, operative treatment was indicated for 62 (24%) e-scooter riders. Orthopaedic surgery was performed more often than maxillofacial surgery. 38 (15%) riders underwent orthopaedic surgery and 24 (10%) maxillofacial surgery (Table 4). While sober riders received orthopaedic surgery in 26 (15%) cases and maxillofacial surgery in 10 (6%), those with alcohol consumption received orthopaedic surgery in 12 (14%) cases and maxillofacial surgery in 14 (17%) cases (Fig. 4).

Non-riders were seen by an orthopaedic trauma surgeon in 94%, by a maxillofacial surgeon in 44% and by neurology/neurosurgery in 13% of cases. 56% of non-riders received standard X-Ray, 19% a CT and 19% a CCT. Surgical wound treatment was performed in 38% and a cast or brace was applied in 31% of cases. Analogue to e-scooter riders, most non-riders (69%) could be discharged from the ED, a direct admission was necessary in 25%, secondary admission in 6% of cases. 13% of non-riders were indicated for 24-h surveillance including vital sign monitoring. The average length of stay was 1.5 (IQR 1.0; 9.5) days for non-riders and highly influenced by one young patient who sustained an open lower arm fracture after falling over an e-scooter as a pedestrian. Operative treatment was indicated in 19% of non-riders, 13% underwent orthopaedic and 6% maxillofacial surgery (Table 4).

### Influence of the mechanism of injury on e-scooter riders

Regarding the mechanism of injury, those e-scooter riders who collided with a stationary or moving object tend to be injured more severely than those who fell without a collision. While most (59%) of the e-scooter riders without a collision presented as “walk-ins” to the ED, those who were in a collision were more likely to present by ambulance/paramedics (56% vs. 34% (*p* = 0.0188)) or emergency physician (9% vs. 3% (ns)). Further, the collision group (41%) was rather triaged as needing immediate or very urgent compared the non-collision group (15%) (*p* = 0.0012). The non-collision group tended to be under the influence of alcohol more often (34% vs. 25%), however the difference was not statistically significant. Even though there was no difference regarding impact to head and face between the non-collision (59%) and collision (56%) groups, ICB (1% vs. 9% (0.0286)) was seen more often in the collision group. Likewise, maxillofacial fractures tended to be more often in the collision group (19% vs 11% (ns)). Fractures of the extremities did not differ in the two groups (25% vs. 26% (ns)). Nevertheless, the e-scooter riders who were in a collision tended to require inpatient treatment (30% vs. 41% (ns)) and operative treatment (23% vs. 35% (ns)) more often.

### Discussion

To our knowledge, this study comprises one of the largest cohorts of e-scooter riders to date and provides the largest cohort of e-scooter-related non-rider injuries studied in Germany to date. To successfully integrate e-scooters into our transportation systems, traffic, and establish carefully weighed rules for their usage, understanding of the associated health risks and related causative factors is essential.

### E-scooter riders vs. non-riders and designated parking areas for e-scooters

Our study establishes that e-scooter riders primarily endanger themselves, as the number of non-rider e-scooter associated injures remains low. However, this aspect must be carefully considered, as non-rider e-scooter associated injuries may be overlooked due to incomplete patient history or documentation. Nevertheless, a large study from Sweden analyzing insurance data from the Swedish Traffic Accident Data Acquisition database (STRADA) included 321 e-scooter associated injuries, where 278 (87%) were e-scooter riders themselves and 43 (13%) were non-riders. Of the 278 e-scooter riders 83% were injured in a crash not involving others^13^. The present study found 92% of e-scooter crashes not involving other parties. According to the STRADA data pedestrians are at the highest risk, either due to collisions with e-scooter riders or from tripping over parked e-scooters^1,3,13^. Similarly, in the present cohort, e-scooter associated non-rider injuries were either from collisions with cyclists or falls over e-scooters placed on sidewalks. While collisions with cyclists and falls over e-scooters were almost equally frequent in our cohort, we did not have any records of a pedestrian who collided with an e-scooter rider. Those pedestrians who tripped over an e-scooter had a median age of 61.2 (IQR 33.9; 83.3) years, and three of seven were 80 years and older, suggesting a higher risk for the elderly. The aspect of pedestrians tripping over parked e-scooters could easily be addressed by establishing stricter parking rules or designated e-scooter parking areas. This would especially protect the vulnerable group of elderly. Only the above mentioned study from Sweden and the present study report collisions with cyclists, which might be due to the lower popularity of bicycles as a daily mode of transportation in other countries, especially in the United States^1,13^.

### Head and facial injuries

In terms of injury patterns sustained from e-scooter usage, our observations match those of earlier studies from different countries^1,3,4,10,11,14^. Body regions especially prone to injuries are the face and upper extremities. Luckily, severe injuries to the head, as well as thoracic or abdominal injuries, are rare. Trivedi et al. examined the high head injury risk, with 38% of injured riders sustaining a minor head injury and 2% an ICH^1^. In this study, alcohol consumption remained an important risk factor for severe head injuries, as 24 (65%) of the 37 patients with a severe head injury or even ICH were intoxicated. None of our patients required neurosurgical intervention. Nevertheless, e-scooter riders should be strongly encouraged to wear a helmet. Studies from Brisbane, Australia, where helmets became mandatory, could clearly demonstrate a reduced overall head injury risk if a helmet was worn^15^.

While maxillofacial trauma and fractures after e-scooter crashes are rather rare (5%) in the study from California, USA, they appear to be more common in European study populations^10,14,16^. In this study, over 50% of e-scooter riders sustained a trauma to the head or face. The higher incidence of severe injuries to the face may be explained by the higher rate of e-scooter riders under the influence of alcohol. While only 5–18% of e-scooter riders in the United States were under the influence of alcohol, European studies report the rate to be between 28 and 37%^1,3,9,10,16^. Shiffler et al. report e-scooter riders in their cohort who sustained craniomaxillofacial trauma were ten times more likely to have been intoxicated than those who did not have craniomaxillofacial injuries^17^. In our study, 48% of the e-scooter riders who hit their head/face and 67% of the patients with maxillofacial fractures were under the influence of alcohol. Our data suggests alcohol to be a major risk factor for injuries to the head and face regions which is further supported by a study conducted with cyclists, stating an impaired ability to ride a bike, even with low blood alcohol concentrations^18^.

### Orthopaedic injuries

Most previous studies on e-scooter injuries only assessed fractures by body region. Graef et al. were the first to give a detailed overview of the fracture types (excluding maxillofacial) and their treatment^11^. However, in their study, which included 43 e-scooter riders, only seven (16%) sustained an extremity fracture and four underwent surgery (radial head plus capitulum, clavicle, tibial plateau, and ankle). The three conservatively treated fractures were two of the radial head and one of the distal radius. We believe that our study cohort of 252 e-scooter riders with 67 extremity fractures and joint dislocations gives a more comprehensive injury pattern overview. We could demonstrate that the upper extremity is more commonly injured than the lower extremity. Studies from the United States and Germany found a comparable distribution with 20–29% of e-scooter riders having fractures of the upper and 5–10% with of fractures of the lower extremities^1,5^. With 14 cases, radial head fractures were the most common entity in our cohort. Most fractures were not dislocated, and operative treatment was only necessary in two cases. Most indications for upper extremity operative treatment were due to fractures of the clavicle, hand, and fingers. In the lower extremity, ankle and tibial plateau fractures were the most common entities and indicated for surgery in six and five cases, respectively. Overall, we found lower extremity fractures to be less common but more severe, as there was one open fracture of the lower leg and two fracture related dislocations, one of the knee and one of the ankle. Even though alcohol seems to be a risk factor for severe injuries to the head and face, patients with fractures or dislocations were intoxicated in only 21% of cases. This is less than the 33% of all e-scooter riders. Nevertheless, 32% of those patients who needed surgery on the extremities were intoxicated, which points to more severe extremity injuries under the influence of alcohol. The lower frequency of injuries to the extremities is most likely caused by an impaired reaction to intercept a fall with the arms or legs and rather falling on the head or face^17,18^.

### Limitations

Even though all data was analyzed meticulously, limitations remain. First, the retrospective design greatly depends on a thoroughly taken history by the treating physician. Second, the patient population is limited to only one city and only one major level 1 trauma center. This may underestimate the real incidence of injuries sustained from e-scooter use as patients presenting at outpatient or urgent care clinics were not recorded^19^. However, since many patients with minor injuries also presented to the ED, we assume that we identified a broad spectrum of e-scooter-related injuries. Further, alcohol breath testing and blood alcohol levels were not obtained routinely and depended on the patients consent. If no breath/blood alcohol level were available, e-scooter riders were only considered as under the influence of alcohol if they were obviously compromised or confirmed relevant alcohol consumption. Accordingly, we do not think that we overestimated the number of riders under the influence of alcohol.

### Conclusion

This study comprises one of the largest cohorts of e-scooter associated injuries to date. Non-rider injuries, especially of the elderly who tripped over e-scooters, may possibly be prevented by considerate parking or designated parking areas. Alcohol consumption is established as a risk factor for more severe and especially injuries to the head and face regions. Therefore, enforcement of drunk driving laws for e-scooters is required and the usage of a helmet must be recommended. These measures could enhance the safety of e-scooters and increase their acceptance as a valuable means of urban transportation.

## Data Availability

All data are available, and requests should be addressed to H.K.
